# A Sensor-Aware Multi-Agent Reinforcement Learning Framework for Joint Data Offloading and Power Control in Edge-Assisted Wireless Sensor Networks

**DOI:** 10.3390/s26123802

**Published:** 2026-06-15

**Authors:** Peiying Zhang, Ruixin Wang, Yuekai Sun, Yujie Yuan

**Affiliations:** 1 Qingdao Institute of Software, College of Computer Science and Technology, China University of Petroleum (East China), Qingdao 266580, China; zhangpeiying@upc.edu.cn (P.Z.); z24070100@s.upc.edu.cn (R.W.); z23070084@s.upc.edu.cn (Y.S.); 2 Shandong Key Laboratory of Intelligent Oil & Gas Industrial Software, Qingdao 266580, China; 3 School of Air Traffic Management, Civil Aviation University of China, Tianjin 300300, China

**Keywords:** wireless sensor networks, mobile edge computing, task offloading, power control, multi-agent reinforcement learning, edge intelligence, sensing data transmission

## Abstract

Wireless sensor networks supported by mobile edge computing are increasingly required to process heterogeneous sensing data under stringent latency, reliability, and energy constraints. However, most existing task-offloading studies are still formulated for generic user equipment and primarily focus on uplink transmission, which is insufficient for practical sensing systems where sensor nodes continuously upload measurements while simultaneously receiving control commands, model updates, and feedback from the edge. To address this gap, this paper reformulates joint computation offloading and power control as a sensor-aware optimization problem in an edge-assisted wireless sensor network. We propose a three-layer architecture consisting of sensor nodes, access points with lightweight edge servers, and a cloud coordination layer. Each sensing task is characterized by data size, computation density, latency deadline, and sensing priority, while the optimization objective jointly minimizes long-term task delay, communication and computation energy, and packet-loss penalty under transmission power, edge resource, and residual-energy constraints. To solve the resulting mixed discrete–continuous problem, we develop a multi-agent reinforcement learning framework in which each sensor node acts as an autonomous agent and learns offloading and transmission policies with clipped proximal policy optimization, while the cloud layer performs coordinated edge-resource allocation through the alternating direction method of multipliers. In addition to delay and energy, network lifetime and sensing delivery performance are incorporated into the evaluation. Simulation results in a sensor-network monitoring scenario demonstrate that the proposed framework consistently reduces latency, lowers energy consumption, and prolongs network lifetime compared with representative baselines, highlighting its effectiveness and practical potential for intelligent sensing applications that require integrated sensing, communication, and edge computing.

## 1. Introduction

Wireless sensor networks (WSNs) and Internet of Things (IoT) sensing systems have become the data-generation front end of modern digital infrastructures [1]. They support a wide range of applications, including environmental monitoring, industrial inspection, intelligent transportation, emergency response, and infrastructure health surveillance. In these applications, large numbers of energy-constrained sensor nodes continuously generate heterogeneous data streams, such as scalar measurements, images, vibration signals, and event-triggered alarms. Although cloud computing provides powerful processing capability, directly uploading all sensing data to remote cloud servers often leads to excessive latency, unstable quality of service, and unnecessary energy expenditure. Mobile edge computing (MEC) offers an effective alternative by moving computation closer to sensing devices and enabling delay-sensitive analytics near the network edge [2].

Nevertheless, directly applying conventional MEC formulations to sensor networks is insufficient, as they fail to capture the sensing-driven characteristics, energy constraints, and bidirectional communication requirements inherent in practical WSNs. Conventional MEC studies mainly focus on communication-oriented devices, while sensor nodes are fundamentally constrained by sensing tasks, residual battery energy, and mission-oriented reliability requirements [3]. Practical sensing systems also involve not only uplink transmission of raw or preprocessed measurements but also downlink dissemination of control commands, model parameters, decision feedback, and reconfiguration instructions [4]. Ignoring downlink overhead may underestimate end-to-end delay and obscure the resource competition between communication and computation. In addition, dense sensor deployments intensify the competition for uplink bandwidth, transmission power, and edge-computing resources under rapidly changing channel conditions. These factors make centralized optimization difficult to scale in practical sensing environments [5].

User-centric or cell-free access architectures provide an attractive foundation for edge-assisted sensor systems [6]. By allowing sensor nodes to be flexibly served by cooperative access points (APs) rather than a single fixed cell, such architectures can improve coverage reliability and alleviate service degradation in edge regions [7]. They also provide favorable support for distributed edge processing in dense wireless sensing environments [8]. However, integrating sensor-aware traffic, bidirectional transmission, and dynamic edge coordination into such architectures introduces a challenging long-term joint optimization problem. The decision process must determine whether a sensing task should be processed locally, partially preprocessed, then offloaded, or fully offloaded. It must also determine how much transmission power should be used and how edge resources should be allocated across competing sensors, while preserving network lifetime and sensing fidelity.

To address these issues, this paper substantially reformulates the original generic MEC problem into a sensor-aware optimization framework for edge-assisted WSNs. Instead of treating devices merely as communication users, we explicitly model them as sensing agents that produce heterogeneous data with different urgency and mission value. We further incorporate bidirectional sensing-related traffic, where uplink channels carry sensing data and downlink channels support edge feedback and control signaling. Building on this formulation, we develop a joint optimization method that combines multi-agent reinforcement learning (MARL) for distributed per-node decision-making [9]. We further employ proximal policy optimization with clipping to stabilize distributed policy learning [10]. At the network side, the alternating direction method of multipliers (ADMM) is used for centralized edge-resource coordination under shared capacity constraints, following the idea of coordinated optimization in user-centric MEC systems [11].

Although energy-aware computation offloading for IoT sensors and WSN-assisted MEC has been studied in prior works [12,13], most existing studies mainly focus on energy-efficient task execution under specific cloudlet, SWIPT, or MEC settings. It should also be noted that power efficiency in WSNs is influenced by multiple factors. For example, node mobility may introduce additional routing overhead and congestion, which affects energy efficiency [14]. Communication strategies such as gossiping-based routing can help reduce redundant transmissions and improve energy usage [15]. In addition, data management mechanisms, including energy-efficient cache placement, also play an important role in reducing transmission cost [16]. In contrast, this paper jointly considers heterogeneous sensing-task priority, bidirectional sensing feedback, residual-energy constraints, packet-loss penalty, uplink power control, and coordinated edge-resource allocation.

This work develops a sensor-aware joint optimization framework for edge-assisted WSNs by incorporating sensing-task priority, bidirectional feedback, residual-energy constraints, packet-loss penalty, and edge-resource competition into the MEC offloading formulation. The resulting mixed discrete–continuous decision problem is addressed through a MARL–ADMM structure, where PPO-Clip enables sensor nodes to learn distributed offloading and power-control policies, and ADMM ensures feasible edge-resource allocation under shared AP-capacity constraints.

The main contributions of this work are summarized as follows.

- We redesign the original MEC framework as a sensor-aware three-layer architecture for edge-assisted WSNs, where sensor nodes, AP-side edge servers, and a cloud coordination layer jointly support sensing-data processing, transmission, and feedback.
- We reformulate task offloading and power control as a joint sensing–communication–computing optimization problem. Unlike conventional MEC studies that treat devices as generic communication users, our formulation explicitly captures sensing-task priority, bidirectional traffic, residual-energy dynamics, and packet-loss penalty in a unified objective. In addition to delay and energy, the objective accounts for sensing priority, packet-loss penalty, and residual-energy constraints, making the model more consistent with practical sensor networks.
- We develop a sensor-aware MARL–ADMM framework for edge-assisted WSNs that is not a trivial integration of existing methods. The MARL layer learns distributed offloading and power-control policies from local observations, avoiding the curse of dimensionality of centralized optimization, while the ADMM layer provides mathematically guaranteed feasible edge-resource allocation under shared AP-capacity constraints. This principled separation of distributed learning and constrained optimization is specifically designed for energy-constrained sensor networks where centralized optimization is infeasible.
- We revise the evaluation from a generic wireless setting to a sensor-network monitoring scenario and assess the proposed method using not only average delay and energy consumption, but also sensing delivery ratio and network lifetime, which are key performance indicators for practical sensing applications.

The remainder of this paper is organized as follows. Section 2 reviews related work on MEC-enabled sensing networks, user-centric edge systems, and reinforcement learning-based offloading. Section 3 presents the sensor-aware system model and problem formulation. Section 4 details the MARL–ADMM solution. Section 5 describes the experimental settings and performance evaluation. Section 6 concludes the paper.

## 2. Related Work

Recent studies on MEC, user-centric networking, and intelligent offloading have provided a useful foundation for edge-assisted sensor systems. This section reviews representative studies in three groups and briefly summarizes their main ideas and limitations.

### 2.1. Task Offloading and Energy-Efficient Edge Computing

Feng et al. [2] presented a comprehensive survey of computation offloading in MEC networks and summarized major design dimensions such as task partitioning, wireless resource allocation, and energy-delay tradeoffs. Their work provides an important general background, but it is not specifically developed for sensing-centric networks with heterogeneous sensing priorities. Zhao et al. [17] investigated energy-saving offloading by jointly allocating radio and computational resources in MEC systems. Their formulation clearly demonstrated the benefit of coordinated communication–computation optimization, yet the model mainly focused on generic MEC users and did not account for sensing-task semantics or node lifetime. Rahmani et al. [18] proposed an energy-efficient task-offloading framework for UAV-enabled MEC with LEO enhancement in Internet of Remote Things networks. Their architecture is highly relevant to remote sensing scenarios, but it emphasizes hierarchical offloading efficiency rather than distributed learning-based decision-making among many competing sensing nodes. Liu et al. [19] studied joint resource allocation and task offloading in MEC through a truthful mechanism design. Their work addressed resource competition in a principled way, although the focus remained on mechanism design rather than the sensing-aware delay, feedback, and battery constraints considered in this paper. Apostolopoulos et al. [20] examined data offloading in UAV-assisted MEC systems under resource uncertainty. Their results highlighted the importance of uncertainty-aware coordination, but the study did not explicitly model bidirectional sensing traffic or user-centric AP cooperation.

### 2.2. User-Centric Networks, Cell-Free Access, and Sensor-Oriented Edge Architectures

Mukherjee and Lee [8] investigated edge-computing-enabled cell-free massive MIMO systems and showed that cell-free architectures can effectively support edge services through distributed access. This work strongly motivates the communication layer of our study, although it does not explicitly formulate sensing-priority-aware offloading. Ammar et al. [6] surveyed user-centric cell-free massive MIMO networks and summarized opportunities, challenges, and possible solutions. Their work offers a broad architectural perspective, but it does not address how sensing tasks should be jointly optimized with edge computation and power control. Wu et al. [21] studied proportional-fair resource allocation in user-centric networks. Their design illustrates the importance of balancing efficiency and fairness in distributed wireless systems, but the objective does not incorporate sensing-task urgency, packet-delivery value, or node residual energy. Lu et al. [22] proposed joint power control and passive beamforming in RIS-assisted user-centric networks. Their results demonstrated that communication quality can be substantially improved by jointly optimizing wireless parameters, which is closely related to our power-control component, although their model does not involve edge task execution. Buzzi et al. [23] compared user-centric 5G cellular networks with the cell-free massive MIMO approach from a resource-allocation perspective. This comparison clarified the advantages of user-centric service structures, but the study remained communication-oriented and did not consider sensing-driven edge intelligence. Ibrahim et al. [24] analyzed the uplink performance of mmWave-fronthaul cell-free massive MIMO systems, while Demirhan and Alkhateeb [25] further showed that wireless millimeter-wave fronthaul can effectively support cell-free systems. These works are useful for understanding high-capacity edge access, but they do not jointly optimize sensing-task offloading and computing allocation. Guenach et al. [26] considered joint power control and AP scheduling in fronthaul-constrained uplink cell-free systems. Their work is closely related to the resource-coupling issue considered here, but it still does not account for sensing-specific QoS or the interaction between task urgency and edge execution.

### 2.3. Learning-Based Offloading and Sensing-Aware Optimization

Schulman et al. [27] proposed TRPO, which stabilizes policy updates by constraining the trust region during learning. Schulman et al. [10] later introduced PPO, which achieves comparable stability with lower implementation complexity and has become a standard baseline for practical policy optimization. Abdolmaleki et al. [28] proposed MPO as a probabilistic policy optimization framework that balances exploration and exploitation. These methods provide the methodological basis for our learning framework. Tampuu et al. [9] demonstrated that deep reinforcement learning can handle multi-agent cooperation and competition in dynamic environments. Their study motivates the use of MARL in our work, where multiple sensing nodes compete for shared wireless and computing resources. Qin et al. [11] developed a decentralized task-offloading and resource-allocation method for user-centric MEC. Their work is one of the closest to ours because it combines user-centric access and learning-based offloading, yet it is not explicitly reformulated for sensing-oriented traffic and sensing-network sustainability. Shen et al. [4] studied robust offloading for edge-computing-assisted sensing and communication systems using deep reinforcement learning. Their work is highly relevant to sensing-oriented edge systems because it explicitly links edge optimization to sensing systems, although it does not consider the joint MARL–ADMM coordination structure proposed here. Yang et al. [29] investigated energy-efficient aerial STAR-RIS-aided computing offloading and content caching for wireless sensor networks. Huang et al. [5] further studied collaborative sensing-aware task offloading and resource allocation for integrated sensing, communication, and computation in intelligent vehicular environments. These recent studies confirm the growing importance of sensing-aware edge optimization, but their application settings and optimization structures differ from the user-centric sensor-network framework considered in this paper. Bi and Zhao [30] proposed a two-layer edge-intelligence architecture for task offloading and computing-capacity allocation with UAV assistance, while Alam et al. [31] optimized joint computational offloading and resource allocation through particle swarm optimization. These works illustrate the diversity of recent optimization approaches, but neither directly addresses the combination of user-centric sensing access, bidirectional sensing feedback, and MARL-based distributed decision-making developed here.

To further clarify the position of this work in the existing literature, Table 1 summarizes representative studies related to sensor-oriented MEC, WSN-assisted edge computing, user-centric edge systems, and learning-based offloading. The comparison focuses on whether each work explicitly considers WSN/IoT sensing scenarios, sensing-task priority, bidirectional communication, power control, edge-resource coordination, and learning-based distributed decision-making.

As shown in Table 1, previous studies have addressed different aspects of energy-efficient offloading, WSN-assisted MEC, user-centric edge systems, and learning-based optimization. However, few of them jointly consider sensing-task priority, bidirectional sensing-related communication, residual-energy-aware operation, packet-delivery penalty, distributed multi-agent decision-making, and coordinated edge-resource allocation. This motivates the sensor-aware MARL–ADMM framework developed in this paper.

Overall, the existing literature suggests that three issues remain insufficiently addressed: sensor-aware task modeling, bidirectional sensing communication, and the joint integration of distributed learning with centralized edge-resource coordination. These gaps motivate the sensor-aware MARL–ADMM framework proposed in this paper.

## 3. Materials and Methods

### 3.1. Sensor-Aware Edge-Assisted Network Architecture

**Figure 1:**
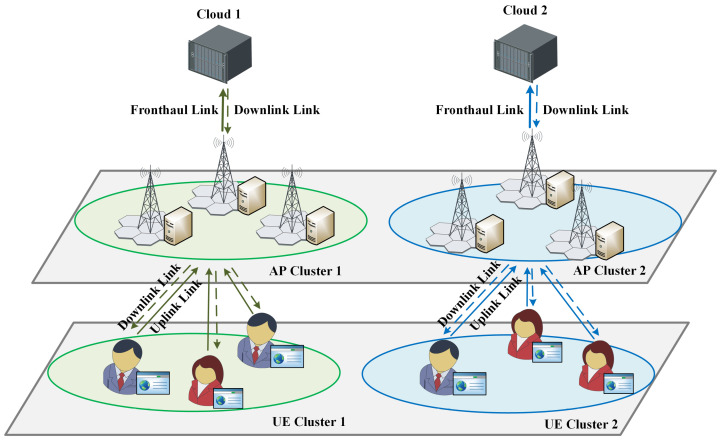
Sensor-aware three-layer edge-assisted network architecture, where sensor nodes upload sensing data to cooperative APs with embedded MEC capability and receive downlink feedback, control commands, or model updates from the edge/cloud layer.

We consider an edge-assisted wireless sensor network composed of three layers: a sensor layer, an AP layer with lightweight MEC capability, and a cloud coordination layer, as illustrated in Figure 1. Let N={1,…,N} denote the set of sensor nodes, M={1,…,M} the set of APs, and K={1,…,K} the set of cloud processors. Each sensor node generates a sensing task periodically or event-driven

(1) $${\mathrm{Task}}_{n}\left(t\right)=\left\{d_{n}\left(t\right),\rho_{n}\left(t\right),D_{n}^{\max}\left(t\right),\omega_{n}\left(t\right)\right\},$$

where $d_{n}\left(t\right)$ is the sensing data size, $\rho_{n}\left(t\right)$ is the required number of CPU cycles per bit, $D_{n}^{\max}\left(t\right)$ is the latency deadline, and $\omega_{n}\left(t\right)$ is the task-priority weight. The priority term is used to distinguish routine monitoring traffic from urgent event-triggered sensing data such as alarms or anomaly snapshots.

In the considered framework, each node has a dual role: sensing and communication. A node may locally preprocess a task, offload it to the nearest cooperative AP cluster, or send it to the cloud through the edge layer if the task requires more computation. The AP layer executes near-real-time sensing analytics such as filtering, fusion, and anomaly detection, while the cloud layer performs global coordination and resource allocation across APs.

### 3.2. Bidirectional Sensing Communication Model

#### 3.2.1. Uplink Sensing Data Transmission

The uplink is used to transmit raw or preprocessed sensing data from node *n* to the serving AP cluster. Let $h_{mn}^{\mathrm{ul}}\left(t\right)$ denote the uplink channel coefficient between node *n* and AP *m* at time slot *t*, including large-scale fading and small-scale fading:

(2) $$h_{mn}^{\mathrm{ul}}\left(t\right)=\sqrt{\beta_{mn}^{\mathrm{ul}}\left(t\right)}\;g_{mn}^{\mathrm{ul}}\left(t\right),$$

where $\beta_{mn}^{\mathrm{ul}}\left(t\right)$ is the large-scale fading coefficient and $g_{mn}^{\mathrm{ul}}\left(t\right)∼\mathrm{CN}\left(0,1\right)$ captures Rayleigh fading. The uplink signal-to-interference-plus-noise ratio (SINR) is

(3) $$\gamma_{n}^{\mathrm{ul}}\left(t\right)=\frac{P_{n}^{\mathrm{ul}}\left(t\right){\left|h_{mn}^{\mathrm{ul}}\left(t\right)\right|}^{2}}{N_{0}B_{\mathrm{ul}}+\sum_{j\neq n}P_{j}^{\mathrm{ul}}\left(t\right){\left|h_{mj}^{\mathrm{ul}}\left(t\right)\right|}^{2}},$$

and the corresponding data rate is

(4) $$r_{n}^{\mathrm{ul}}\left(t\right)=\eta_{\mathrm{ul}}B_{\mathrm{ul}}\log_{2}\;\left(1+\gamma_{n}^{\mathrm{ul}}\left(t\right)\right),$$

where $P_{n}^{\mathrm{ul}}\left(t\right)$ is the transmit power selected by node *n*, $N_{0}$ is the noise power spectral density, $B_{\mathrm{ul}}$ is the uplink bandwidth, and $\eta_{\mathrm{ul}}$ is the effective bandwidth utilization factor.

#### 3.2.2. Downlink Feedback and Control Transmission

The downlink carries edge-generated responses, control instructions, model updates, or sensing-parameter reconfiguration messages. Let $h_{mn}^{\mathrm{dl}}\left(t\right)$ denote the downlink channel coefficient. The downlink SINR is written as

(5) $$\gamma_{n}^{\mathrm{dl}}\left(t\right)=\frac{P_{m}^{\mathrm{dl}}\left(t\right){\left|h_{mn}^{\mathrm{dl}}\left(t\right)\right|}^{2}}{N_{0}B_{\mathrm{dl}}+I_{n}^{\mathrm{dl}}\left(t\right)},$$

where $P_{m}^{\mathrm{dl}}\left(t\right)$ is the AP downlink power and $I_{n}^{\mathrm{dl}}\left(t\right)$ denotes aggregate downlink interference. The downlink rate is

(6) $$r_{n}^{\mathrm{dl}}\left(t\right)=\eta_{\mathrm{dl}}B_{\mathrm{dl}}\log_{2}\;\left(1+\gamma_{n}^{\mathrm{dl}}\left(t\right)\right).$$

This bidirectional design is more suitable for sensor systems than uplink-only models because sensing decisions often depend on timely feedback from the edge, which is increasingly emphasized in recent sensing-aware edge studies [4,5].

### 3.3. Latency Model

The end-to-end completion delay of sensing task *n* includes local preprocessing, uplink transmission, edge computation, and downlink feedback:

(7) $$D_{n}^{\mathrm{tot}}\left(t\right)=D_{n}^{\mathrm{pre}}\left(t\right)+\delta_{n}\left(t\right)\left(D_{n}^{\mathrm{ul}}\left(t\right)+D_{n}^{\mathrm{edge}}\left(t\right)+D_{n}^{\mathrm{dl}}\left(t\right)\right)+\left(1-\delta_{n}\left(t\right)\right)D_{n}^{\mathrm{loc}}\left(t\right),$$

where $\delta_{n}\left(t\right)\in \left\{0,1\right\}$ is the offloading decision. Specifically,

(8) $$\begin{array}{cc} D_{n}^{\mathrm{pre}}\left(t\right) & =\frac{\kappa_{n}d_{n}\left(t\right)}{f_{n}^{\mathrm{pre}}\left(t\right)}, \end{array}$$

(9) $$\begin{array}{cc} D_{n}^{\mathrm{ul}}\left(t\right) & =\frac{d_{n}\left(t\right)}{r_{n}^{\mathrm{ul}}\left(t\right)}, \end{array}$$

(10) $$\begin{array}{cc} D_{n}^{\mathrm{edge}}\left(t\right) & =\frac{d_{n}\left(t\right)\rho_{n}\left(t\right)}{c_{n}^{\mathrm{edge}}\left(t\right)}, \end{array}$$

(11) $$\begin{array}{cc} D_{n}^{\mathrm{dl}}\left(t\right) & =\frac{d_{n}^{\mathrm{fb}}\left(t\right)}{r_{n}^{\mathrm{dl}}\left(t\right)}, \end{array}$$

(12) $$\begin{array}{cc} D_{n}^{\mathrm{loc}}\left(t\right) & =\frac{d_{n}\left(t\right)\rho_{n}\left(t\right)}{f_{n}^{\mathrm{loc}}\left(t\right)}. \end{array}$$

Here, $\kappa_{n}$ is the preprocessing ratio, $f_{n}^{\mathrm{pre}}\left(t\right)$ is the preprocessing frequency, $c_{n}^{\mathrm{edge}}\left(t\right)$ is the edge-computing resource allocated to node *n*, $d_{n}^{\mathrm{fb}}\left(t\right)$ is the size of feedback data, and $f_{n}^{\mathrm{loc}}\left(t\right)$ is the local CPU frequency.

### 3.4. Energy and Network-Lifetime Model

Compared with the original MEC model, we explicitly distinguish sensing-related energy from communication energy. The total energy consumption of node *n* is

(13) $$E_{n}^{\mathrm{tot}}\left(t\right)=E_{n}^{\mathrm{sense}}\left(t\right)+E_{n}^{\mathrm{pre}}\left(t\right)+E_{n}^{\mathrm{tx}}\left(t\right)+E_{n}^{\mathrm{loc}}\left(t\right),$$

where $E_{n}^{\mathrm{sense}}\left(t\right)$ is the sensing energy required to acquire data, $E_{n}^{\mathrm{pre}}\left(t\right)$ is the local preprocessing energy, $E_{n}^{\mathrm{tx}}\left(t\right)=P_{n}^{\mathrm{ul}}\left(t\right)D_{n}^{\mathrm{ul}}\left(t\right)$ is the transmission energy, and $E_{n}^{\mathrm{loc}}\left(t\right)$ is the local execution energy when the task is not offloaded. Let $B_{n}\left(t\right)$ denote the residual battery energy:

(14) $$B_{n}\left(t+1\right)=B_{n}\left(t\right)-E_{n}^{\mathrm{tot}}\left(t\right)+E_{n}^{\mathrm{harv}}\left(t\right),$$

where $E_{n}^{\mathrm{harv}}\left(t\right)$ denotes optional harvested energy. The network lifetime is evaluated by the first-node-death time or, equivalently, by the persistence of all nodes above a safety battery threshold $B_{\min}$.

### 3.5. Optimization Objective

Our objective is to jointly minimize long-term sensing delay, total energy consumption, and packet-loss penalty while maintaining residual-energy feasibility. The problem is

(15) $$\min_{\left\{\delta_{n}\left(t\right),P_{n}^{\mathrm{ul}}\left(t\right),P_{m}^{\mathrm{dl}}\left(t\right),c_{n}^{\mathrm{edge}}\left(t\right)\right\}}\frac{1}{T}\sum_{t=1}^{T}\sum_{n=1}^{N}\left[\alpha \omega_{n}\left(t\right)D_{n}^{\mathrm{tot}}\left(t\right)+\beta E_{n}^{\mathrm{tot}}\left(t\right)+\zeta L_{n}\left(t\right)\right],$$

subject to

(16) $$\begin{array}{cc} D_{n}^{\mathrm{tot}}\left(t\right) & \leq D_{n}^{\max}\left(t\right), \end{array}$$

(17) $$\begin{array}{cc} 0\leq P_{n}^{\mathrm{ul}}\left(t\right) & \leq P_{\max}^{\mathrm{ul}}, \end{array}$$

(18) $$\begin{array}{cc} 0\leq P_{m}^{\mathrm{dl}}\left(t\right) & \leq P_{\max}^{\mathrm{dl}}, \end{array}$$

(19) $$\begin{array}{cc} \sum_{n\in N_{m}}c_{n}^{\mathrm{edge}}\left(t\right) & \leq C_{m}, \end{array}$$

(20) $$\begin{array}{cc} B_{n}\left(t\right) & \geq B_{\min}, \end{array}$$

for all n,m,t. Here, $L_{n}\left(t\right)$ denotes the packet-loss or delivery-failure penalty, and α, β, and ζ are weight coefficients. This formulation adopts a sensing-oriented metric set and is conceptually consistent with recent sensing-aware offloading formulations [5,29].

## 4. Proposed MARL–ADMM Algorithm

### 4.1. Multi-Agent Reinforcement Learning for Sensor Nodes

Each sensor node is modeled as an autonomous agent that observes local sensing, channel, and battery states and outputs an offloading and power-control decision. The observation of node *n* at time slot *t* is defined as

(21) $$s_{n}\left(t\right)=\left[\gamma_{n}^{\mathrm{ul}}\left(t\right),\gamma_{n}^{\mathrm{dl}}\left(t\right),d_{n}\left(t\right),\omega_{n}\left(t\right),B_{n}\left(t\right),Q_{m}\left(t\right),{\bar{D}}_{n}\left(t\right)\right],$$

where $Q_{m}\left(t\right)$ is the queue state of the serving AP and ${\bar{D}}_{n}\left(t\right)$ is the historical average delay.

The action of sensor node *n* is defined as

(22) $$a_{n}\left(t\right)=\left[\delta_{n}\left(t\right),\;P_{n}^{\mathrm{ul}}\left(t\right),\;\nu_{n}\left(t\right)\right],$$

where $\delta_{n}\left(t\right)\in \left\{0,1\right\}$ denotes the binary offloading decision, $P_{n}^{\mathrm{ul}}\left(t\right)\in \left[0,P_{\max}^{\mathrm{ul}}\right]$ denotes the uplink transmission power, and $\nu_{n}\left(t\right)\in \left[0,1\right]$ denotes the requested edge-resource ratio.

The PPO actor network outputs a hybrid action distribution for the mixed discrete–continuous action space. Specifically, the offloading decision $\delta_{n}\left(t\right)$ is sampled from a Bernoulli distribution. For the requested edge-resource ratio, the actor network generates two positive parameters through a softplus activation, and $\nu_{n}\left(t\right)$ is sampled from a Beta distribution:

(23) $$\nu_{n}\left(t\right)∼\mathrm{Beta}\left(\alpha_{n}\left(t\right),\beta_{n}\left(t\right)\right),$$

where $\alpha_{n}\left(t\right)>0$ and $\beta_{n}\left(t\right)>0$ are the distribution parameters produced by the actor network. During training, $\nu_{n}\left(t\right)$ is sampled from the Beta distribution to encourage exploration. During evaluation, the deterministic mean of the distribution is used:

(24) $$\nu_{n}\left(t\right)=\frac{\alpha_{n}\left(t\right)}{\alpha_{n}\left(t\right)+\beta_{n}\left(t\right)}.$$

This design allows each sensor node to express a flexible resource demand while ensuring that the requested ratio remains within the feasible interval [0,1].

Before ADMM-based coordination, each AP first collects the offloading decisions and requested resource ratios from its associated sensor nodes. For AP *m*, the raw requested edge-computing demand of sensor node *n* is calculated as

(25) $${\hat{c}}_{n}\left(t\right)=\delta_{n}\left(t\right)\nu_{n}\left(t\right)C_{m},\;n\in N_{m}.$$

When the aggregate requested demand does not exceed the AP capacity, the requests are preserved. When the aggregate requested demand exceeds the available AP capacity, the AP constructs a feasible initial allocation by proportional normalization:

(26) $$c_{n}^{\left(0\right)}\left(t\right)=\left\{\begin{array}{cc} {\hat{c}}_{n}\left(t\right), & \sum_{j\in N_{m}}{\hat{c}}_{j}\left(t\right)\leq C_{m},\\ \frac{{\hat{c}}_{n}\left(t\right)}{\sum_{j\in N_{m}}{\hat{c}}_{j}\left(t\right)}C_{m},\; & \sum_{j\in N_{m}}{\hat{c}}_{j}\left(t\right)>C_{m}. \end{array}\right.$$

The normalized allocation $c_{n}^{\left(0\right)}\left(t\right)$ is then used as the feasible initialization for the subsequent ADMM-based resource-allocation procedure.

PPO-Clip is adopted to improve training stability in this multi-agent environment [10]. For each sensor agent, both the actor and critic are implemented as two-layer multilayer perceptrons with ReLU activation functions. The actor network outputs the parameters of the hybrid action distribution, while the critic network estimates the scalar state-value function.

The reward function is constructed to reflect sensing-specific objectives:

(27) $$r_{n}\left(t\right)=-\alpha \omega_{n}\left(t\right)D_{n}^{\mathrm{tot}}\left(t\right)-\beta E_{n}^{\mathrm{tot}}\left(t\right)-\zeta L_{n}\left(t\right)+\lambda_{\mathrm{life}}I\left[B_{n}\left(t\right)\geq B_{\min}\right],$$

where $\lambda_{\mathrm{life}}$ is a positive reward associated with preserving residual energy and I[·] is the indicator function. This reward explicitly discourages strategies that reduce short-term delay at the expense of sensor lifetime.

### 4.2. ADMM-Based Edge-Resource Coordination

Given the requested offloading loads from all agents, the cloud layer coordinates edge-computing allocations by solving

(28) $$\min_{\left\{c_{n}^{\mathrm{edge}}\left(t\right)\right\}}\sum_{m=1}^{M}\sum_{n\in N_{m}}{\left(c_{n}^{\mathrm{edge}}\left(t\right)-{\hat{c}}_{n}\left(t\right)\right)}^{2}$$

subject to the AP-capacity constraints. For AP *m*, define $c_{m}\left(t\right)={\left\{c_{n}^{\mathrm{edge}}\left(t\right)\right\}}_{n\in N_{m}}$ and ${\hat{c}}_{m}\left(t\right)={\left\{{\hat{c}}_{n}\left(t\right)\right\}}_{n\in N_{m}}$. The feasible resource-allocation set is given by

(29) $$C_{m}=\left\{c_{m}\left(t\right)|c_{n}^{\mathrm{edge}}\left(t\right)\geq 0,\;\sum_{n\in N_{m}}c_{n}^{\mathrm{edge}}\left(t\right)\leq C_{m}\right\}.$$

To solve the above quadratic allocation problem, we introduce an auxiliary variable $z_{m}\left(t\right)$ for each AP and rewrite the problem as

(30) $$\min_{\left\{c_{m},z_{m}\right\}}\sum_{m=1}^{M}{∥c_{m}\left(t\right)-{\hat{c}}_{m}\left(t\right)∥}_{2}^{2}+\sum_{m=1}^{M}I_{C_{m}}\left(z_{m}\left(t\right)\right),\;s.t.\;c_{m}\left(t\right)-z_{m}\left(t\right)=0,$$

where $I_{C_{m}}\left(\cdot \right)$ is the indicator function of the feasible set $C_{m}$. Using the scaled dual variable $u_{m}\left(t\right)$, the ADMM updates at iteration *k* are given by

(31) $$c_{m}^{k+1}\left(t\right)=\frac{2{\hat{c}}_{m}\left(t\right)+\rho \left(z_{m}^{k}\left(t\right)-u_{m}^{k}\left(t\right)\right)}{2+\rho },$$

(32) $$z_{m}^{k+1}\left(t\right)=\Pi_{C_{m}}\left(c_{m}^{k+1}\left(t\right)+u_{m}^{k}\left(t\right)\right),$$

(33) $$u_{m}^{k+1}\left(t\right)=u_{m}^{k}\left(t\right)+c_{m}^{k+1}\left(t\right)-z_{m}^{k+1}\left(t\right),$$

where $\Pi_{C_{m}}\left(\cdot \right)$ denotes the Euclidean projection onto $C_{m}$. For a vector $v_{m}$, this projection is computed as

(34) $$\Pi_{C_{m}}\left(v_{m}\right)={\left[v_{m}-\tau_{m}1\right]}^{+},$$

where ${\left[\cdot \right]}^{+}=\max\left(\cdot ,0\right)$ is applied element-wise. If $\sum_{n\in N_{m}}{\left[v_{n}\right]}^{+}\leq C_{m}$, then $\tau_{m}=0$; otherwise, $\tau_{m}$ is chosen such that

(35) $$\sum_{n\in N_{m}}{\left[v_{n}-\tau_{m}\right]}^{+}=C_{m}.$$

A fixed penalty parameter is used for ADMM, and its value is set to ρ=1.0 in all simulations. The primal and dual residuals are defined as

(36) $$r_{m}^{k+1}\left(t\right)=c_{m}^{k+1}\left(t\right)-z_{m}^{k+1}\left(t\right),$$

(37) $$s_{m}^{k+1}\left(t\right)=\rho \left(z_{m}^{k+1}\left(t\right)-z_{m}^{k}\left(t\right)\right).$$

The ADMM iterations terminate when

(38) $$\max_{m}{∥r_{m}^{k+1}\left(t\right)∥}_{2}\leq \epsilon_{\mathrm{pri}},\;\max_{m}{∥s_{m}^{k+1}\left(t\right)∥}_{2}\leq \epsilon_{\mathrm{dual}},$$

or when the maximum number of ADMM iterations is reached. In the simulations, $\epsilon_{\mathrm{pri}}=\epsilon_{\mathrm{dual}}=10^{-3}$ and the maximum number of ADMM iterations is set to 50. The ADMM resource-allocation procedure is executed once in each decision slot after the sensor agents generate their offloading and resource-request actions.

### 4.3. Integrated Operation

The complete optimization procedure operates as follows:

1. Each sensor node senses its local state and generates an action through the PPO-based policy network.
2. The proposed action is projected onto the feasible region defined by power and energy constraints.
3. APs aggregate offloading requests and forward resource demands to the cloud coordinator.
4. The cloud solves the ADMM resource-allocation subproblem and returns edge-resource decisions.
5. Sensor nodes receive feedback, update task execution, observe rewards, and continue policy learning.

### 4.4. Complexity and Convergence Analysis

The proposed MARL–ADMM framework consists of two computational components: PPO-based policy learning at the sensor-agent side and ADMM-based edge-resource coordination at the cloud or AP coordination layer. This subsection discusses the computational complexity and convergence properties of these two components.

For the PPO component, the training complexity depends on the number of sensor agents, the number of sampled transitions, the number of PPO update epochs, and the size of the actor–critic networks. Let *N* denote the number of sensor nodes, *B* the batch size, $E_{\mathrm{ppo}}$ the number of PPO update epochs per training iteration, and $C_{\theta }$ the computational cost of one forward–backward update of the actor–critic networks. The complexity of one PPO training iteration can be approximated as

(39) $$O\left(NE_{\mathrm{ppo}}BC_{\theta }\right).$$

If parameter sharing is adopted among homogeneous sensor agents, the number of trainable network parameters does not increase linearly with *N*, although the sampling and inference cost still increases with the number of active sensor nodes.

During online execution, each sensor node only performs a forward pass through the trained actor network. Let $C_{\pi }$ denote the computational cost of one actor–network inference. The online policy-inference cost of all sensor nodes in one decision slot is therefore

(40) $$O\left(NC_{\pi }\right).$$

This is substantially lighter than solving the original mixed discrete–continuous optimization problem at each sensor node and is therefore more suitable for energy-constrained WSN deployment.

For the ADMM component, the resource-allocation subproblem is executed after sensor agents generate their offloading and resource-request actions. For AP *m*, let $|N_{m}|$ denote the number of associated sensor nodes and $K_{\mathrm{admm}}$ denote the maximum number of ADMM iterations. Since the edge-resource allocation problem has a quadratic objective and linear AP-capacity constraints, the main computational cost in each ADMM iteration comes from the primal update and the projection onto the feasible AP-resource set. Therefore, the complexity of ADMM coordination at AP *m* can be approximated as

(41) $$O\left(K_{\mathrm{admm}}|N_{m}\left|\log\right|N_{m}|\right),$$

where the logarithmic term is associated with projection onto the box-constrained simplex. Across all APs, the total coordination complexity is

(42) $$O\left(\sum_{m=1}^{M}K_{\mathrm{admm}}\left|N_{m}\right|\log\left|N_{m}\right|\right).$$

Regarding convergence, the PPO-based MARL component is a stochastic policy-gradient method applied to a non-convex multi-agent learning problem. Therefore, the global optimality of the overall long-term stochastic control problem cannot be theoretically guaranteed. Nevertheless, PPO-Clip improves empirical training stability by restricting the policy update ratio within a clipped interval, which prevents excessively large policy updates during learning.

In contrast, the ADMM-based edge-resource allocation subproblem is convex under the quadratic objective and linear AP-capacity constraints. Specifically, for a fixed set of PPO-generated resource requests, the per-slot resource-allocation problem minimizes a convex quadratic function over a closed and convex feasible set. Under standard ADMM assumptions, including convexity, closedness, and nonempty feasibility of the constraint set, the ADMM iterations converge to a primal-dual optimal solution of the per-slot resource-allocation subproblem. Therefore, although the full MARL–ADMM framework does not guarantee global optimality for the original non-convex long-term decision problem, the ADMM layer guarantees feasibility and convergence for the edge-resource coordination problem in each decision slot.

## 5. Results

### 5.1. Experimental Setup

The proposed sensor-aware MEC framework is evaluated through extensive simulations using a hybrid simulation platform. OMNeT++ is employed for network-level simulation, while Python 3.8 with PyTorch 1.13 is used to implement the proposed MARL–ADMM algorithm. The test environment utilizes an NVIDIA GeForce RTX 3080 GPU and a 12-core Intel Xeon E5-2680 v4 CPU @ 2.4 GHz with 96 GB RAM. Table 2 summarizes the key simulation parameters derived from the system model in Section 3.

The simulation scenario considers an edge-assisted wireless sensor network deployed in a 900×900 m^2^ monitoring area with 3 CPUs positioned at fixed locations. Sensor nodes randomly distributed in the sensing field periodically upload sensed data and may additionally generate event-triggered sensing tasks under abnormal conditions. The average sensing-task arrival rate is set to $\lambda_{t}=0.8$ tasks/s/node. Each task requires [100, 200] KB data transmission and [1000, 2000] cycles/bit computation density. In this way, the simulation emulates practical sensing scenarios such as environmental monitoring, industrial condition sensing, and anomaly-aware edge intelligence, where both sensing data upload and downlink feedback are involved in the task execution process.

### 5.2. Comparison Schemes

To evaluate the effectiveness of the proposed optimization scheme, the following benchmark methods are implemented for comparative analysis:

- **Clipped Objective Policy Optimization (CBO)** [17]: A clipped-policy-based learning benchmark adopted for comparison with the proposed method under similar policy-constrained optimization logic.
- **Trust Region Policy Optimization (TRPO)** [27]: TRPO iteratively updates a stochastic policy by maximizing a surrogate objective subject to a KL-divergence constraint, ensuring stable policy improvement.
- **Maximum a Posteriori Policy Optimization (MPO)** [28]: MPO optimizes policies using a probabilistic update mechanism that balances exploration and exploitation according to observed rewards.
- **Local Processing (LP)**: All sensing tasks are processed locally at the sensor nodes, thus avoiding communication overhead while suffering from limited on-device computation capability.
- **All Offloading (AO)**: All sensing tasks are offloaded to the edge/cloud side, which may reduce local processing burden but often causes transmission congestion and resource mismatch under dense deployments.
- **Sensing-Aware Offloading (SAO)** [5]: This scheme performs collaborative sensing-aware task offloading by jointly considering sensing-task processing and communication-computing resource allocation. It can effectively improve the efficiency of sensing-oriented task execution, but its adaptability may be limited under highly dynamic network conditions.
- **Multi-Agent Proximal Policy Optimization (MAPPO)** [32]: MAPPO is an on-policy multi-agent reinforcement learning method with centralized training and decentralized execution and is adopted as a representative cooperative MARL baseline for dynamic task offloading and resource allocation. The actor and critic networks use the same MLP architecture (128–64, ReLU) as the proposed method, with a learning rate of $5\times 10^{-4}$, discount factor 0.99, clip ratio 0.2, GAE 0.95, and batch size 256.
- **Multi-Agent Deep Deterministic Policy Gradient (MADDPG)** [33]: MADDPG is an off-policy actor–critic MARL algorithm designed for continuous multi-agent decision-making and is used to evaluate the proposed method against a deterministic policy-gradient-based baseline. The actor network uses an MLP with 128–64 hidden units (ReLU), and the critic network uses 256–128 hidden units (ReLU). The learning rate is $1\times 10^{-3}$, with a replay buffer of size $10^{5}$, batch size 128, and OU exploration noise with σ=0.1.
- **Graph Reinforcement Learning (Graph RL)** [34]: Graph RL combines graph-based representation learning with reinforcement learning to capture task or network dependencies and is selected as a topology-aware learning baseline for task offloading. It uses a GCN with two graph convolution layers (hidden dimension 64) followed by an MLP (64–32, ReLU). The learning rate is $5\times 10^{-4}$, and the discount factor is 0.99.
- **Federated Reinforcement Learning (Federated RL)** [35]: Federated RL integrates federated learning with reinforcement learning to support distributed policy learning without sharing raw local data and is used as a privacy-preserving distributed learning baseline. PPO training is distributed across sensor nodes, with FedAvg aggregation every 20 episodes and a participation ratio of 0.8. The local PPO hyperparameters are identical to those of the proposed method.

### 5.3. Performance Evaluation

**Figure 2:**
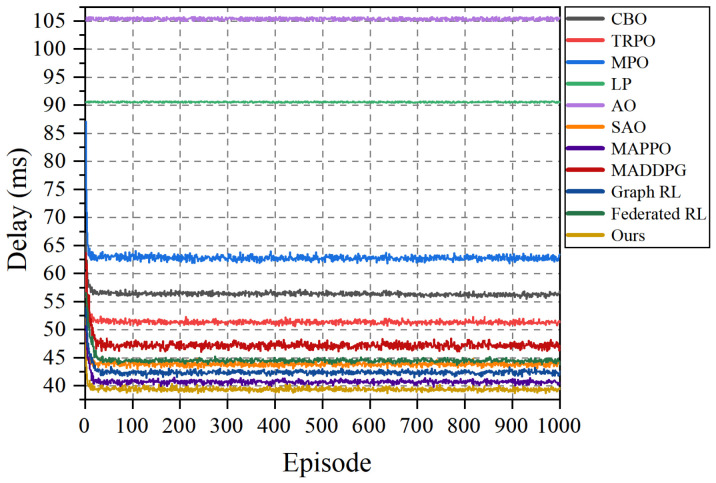
Comparison of sensing-task delay across different schemes.

**Figure 3:**
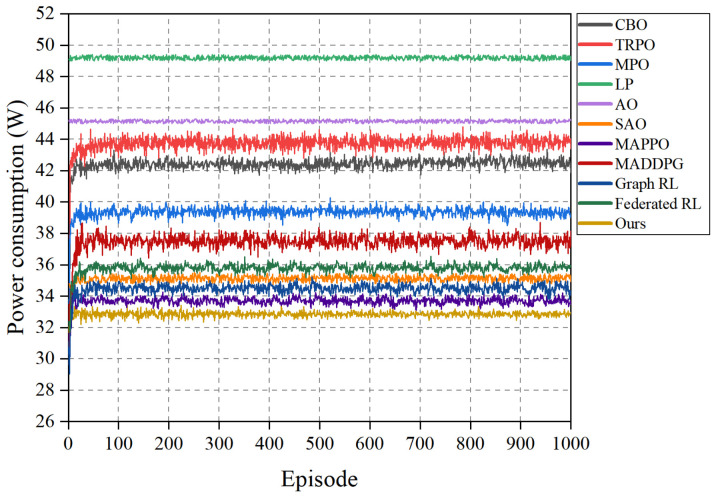
Comparison of power consumption across different schemes.

**Figure 4:**
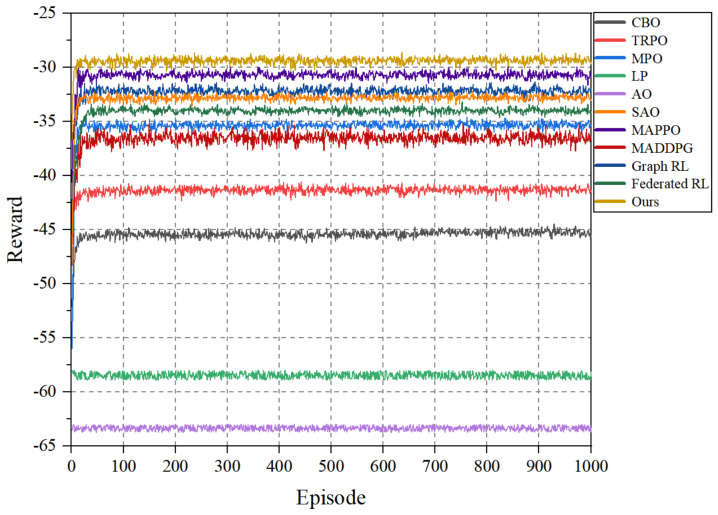
Comparison of cumulative reward across different schemes.

To evaluate the reproducibility and statistical stability of the experimental results, all compared methods were independently executed over five random seeds under the same simulation configuration. In each run, the neural-network initialization, task-arrival process, wireless channel condition, and sensor–node deployment were randomly regenerated. Therefore, the learning curves in Figure 2, Figure 3 and Figure 4 represent the averaged results over independent runs rather than a single deterministic trial.

To evaluate the reproducibility and statistical stability of the experimental results, all compared methods were independently executed over five random seeds (28, 42, 1024, 2025, and 3407) under the same simulation configuration. All baselines were trained and evaluated under identical simulation settings, including the same task-arrival process, channel conditions, and sensor–node deployment, differing only in the offloading and power-control algorithm. In each run, the neural-network initialization, task-arrival process, wireless channel condition, and sensor–node deployment were randomly regenerated. Therefore, the learning curves in Figure 2, Figure 3 and Figure 4 represent the averaged results over independent runs rather than a single deterministic trial.

Table 3 further reports the statistical results of delay, power consumption, and cumulative reward, including the mean value, standard deviation, 95% confidence interval, and *p*-value compared with the proposed MARL–ADMM framework. The *p*-values are computed using a two-tailed independent two-sample t-test that compares each baseline method against the proposed MARL–ADMM framework under the null hypothesis that the two methods have equal means. For delay and power consumption, lower values indicate better performance, while for cumulative reward, higher values are preferred.

The statistical results in Table 3 provide quantitative evidence for the stability and reproducibility of the proposed method. The detailed performance comparisons are discussed in the following subsections according to task delay, power consumption, and cumulative reward, respectively.

In addition, to complement the theoretical convergence discussion, we measure the empirical convergence behavior of the ADMM component used for edge-resource coordination. The ADMM layer achieves convergence within $K_{\mathrm{admm}}=15$ iterations on average (maximum 25 iterations) across all evaluation time slots. The average computation time for ADMM coordination per time slot is 4.2 ms. Across all 50 APs, the total coordination time remains below 12 ms per slot, which is well within typical sensing-task latency deadlines (100–500 ms in the evaluated scenario).

#### 5.3.1. Task Delay

As illustrated by the sensing-task delay comparison in Figure 2, the MARL–ADMM framework minimizes latency while exhibiting stable training convergence. The statistical evidence underscores its capacity to buffer dynamic workloads and coordinate edge resources effectively. Crucially, this latency reduction does not come at the expense of other metrics; instead, the framework maintains a co-optimized balance across power consumption and cumulative reward.

The MARL agents adaptively determine offloading and power-control strategies according to workload variations and channel conditions, while the ADMM-based coordination layer efficiently allocates edge-side resources among APs. Consequently, the proposed framework effectively reduces queue accumulation and alleviates resource mismatch under dynamic sensing scenarios, leading to more stable and efficient delay performance.

#### 5.3.2. Power Consumption

Figure 3 confirms that the MARL–ADMM framework yields the lowest average power consumption. This efficiency stems from its adaptive offloading and power-control policies, which eliminate superfluous transmission overhead and local computational redundancy.

The improvement is mainly attributed to the interaction between local policy learning and ADMM-based resource coordination. The MARL agents adjust their actions according to workload and channel states, while the ADMM layer mitigates resource conflicts among APs. As a result, the proposed method avoids both excessive local computation and inefficient full offloading, leading to more energy-efficient sensing-task execution.

#### 5.3.3. Reward

Figure 4 shows that the proposed MARL–ADMM framework achieves the highest cumulative reward. Since the reward jointly reflects task delay, power consumption, and queue stability, this result demonstrates that the proposed method optimizes the overall system objective rather than a single performance metric.

The reward gain confirms the benefit of combining MARL-based local decision-making with ADMM-based global coordination. While some baselines may perform well on one metric, they usually sacrifice energy efficiency or long-term reward. In contrast, the proposed framework achieves a better balance among latency, energy consumption, and sensing-task execution efficiency.

### 5.4. Ablation Study

To further validate the effectiveness of each design component in the proposed sensor-aware MARL–ADMM framework, we conduct an ablation study in the context of edge-assisted wireless sensor networks. In practical sensing applications, the end-to-end performance depends not only on task offloading itself but also on coordinated resource allocation, bidirectional transmission modeling, and task-aware reward design. Therefore, we consider the following ablated variants: (1) removing the ADMM-based coordination module, (2) removing downlink transmission modeling, (3) removing the queue-aware reward term, and (4) removing the task-priority mechanism. The corresponding results are summarized in Table 4.

Table 4 demonstrates that the complete framework consistently outperforms all ablated variants across all metrics. Notably, omitting the ADMM-based coordination module triggers the most severe performance degradation, with a 26.0% increase in delay and a 13.0% increase in power consumption. This highlights that global coordination among APs and cloud processors is indispensable in dense network deployments, where uncoordinated local policies inevitably exacerbate channel contention and resource mismatch. Without such coordination, local decisions made by agents are more likely to cause queue accumulation and resource mismatch at the edge layer.

Similarly, excluding downlink transmission modeling also leads to a noticeable increase in end-to-end delay of 19.1% and a 9.0% rise in power consumption. This result is especially important from the perspective of practical sensing applications because sensing systems usually involve not only uplink data reporting but also downlink feedback, control instructions, acknowledgment signaling, or lightweight model updates. Ignoring the downlink part causes the framework to underestimate the actual service latency of sensing tasks and may produce overly optimistic offloading decisions.

When the queue-aware reward term is removed, both delay and reward deteriorate, with a 12.8% increase in delay and a 5.7% increase in power consumption. This demonstrates that queue-state information plays a key role in stabilizing the sensing workload across APs, especially when event-triggered sensor traffic bursts occur. In addition, removing task-priority awareness also weakens the overall performance, resulting in a 7.1% delay increase. Since sensing applications often contain heterogeneous tasks with different urgency levels, such as routine environmental monitoring and emergency anomaly reporting, task-priority modeling is beneficial for protecting delay-sensitive sensing information.

Overall, the ablation study confirms that the performance improvement of the proposed framework does not arise from a single module. Instead, it is the result of the joint effect of hierarchical coordination, bidirectional sensing-communication modeling, and sensor-aware reward shaping, with ADMM-based global coordination and downlink modeling being the most critical contributors.

### 5.5. Sensitivity Analysis

To further examine the adaptability of the proposed framework to different wireless sensing environments, we conduct a sensitivity analysis from three aspects: the number of sensor nodes, the sensing-task arrival rate, and the reward weights. These factors are highly relevant to practical sensing scenarios, where node density, sensing frequency, and system objectives may vary significantly across application domains such as industrial monitoring, intelligent transportation, and environmental surveillance.

#### 5.5.1. Impact of the Number of Sensor Nodes

We first investigate the impact of sensor–node density on the proposed framework. Table 5 reports the performance under different numbers of sensor nodes.

It can be seen from Table 5 that as the number of sensor nodes increases, the average delay and power consumption gradually rise, while the cumulative reward decreases. Notably, the tightly bounded standard deviations across scales confirm the framework’s operational consistency. Although dense deployments intensify intra-cell interference and computational contention, the resultant performance degradation remains marginal. This scalability validates the framework’s viability for high-density WSNs characterized by concurrent, large-scale data generation.

#### 5.5.2. Impact of Task-Arrival Rate

We then evaluate the influence of the sensing-task arrival rate. In practical sensor systems, task arrivals may vary from periodic low-rate reporting to bursty event-driven uploads. Table 6 summarizes the corresponding results.

As the task-arrival rate increases, both delay and power consumption rise, whereas the cumulative reward decreases. The low variance within each workload tier indicates that our model effectively tracks dynamic traffic changes with robust empirical consistency. This indicates that heavier sensing workloads lead to higher communication contention and more severe queue accumulation at edge servers. Nevertheless, the proposed framework still exhibits relatively stable performance under moderate and high traffic loads, which demonstrates its ability to adapt to dynamic sensing workloads. This feature is particularly important for sensing scenarios involving event-triggered reporting, where sudden bursts of sensing traffic may occur due to detected abnormalities or environmental changes.

#### 5.5.3. Impact of Reward Weights

Finally, we analyze the sensitivity of the proposed framework to different reward weights associated with delay and energy consumption. Since different sensing applications may have different operational priorities, such as timeliness in emergency detection or energy efficiency in long-term environmental monitoring, this analysis is necessary to demonstrate the flexibility of the proposed framework. The results are shown in Table 7.

It can be observed that when a larger weight is assigned to delay, the framework tends to adopt more aggressive offloading and transmission decisions, thereby achieving lower latency at the cost of slightly higher power consumption. The stable and small standard deviations across all resource ratios confirm that the trade-off optimization results are highly reliable and repeatable. In contrast, when energy is emphasized, the framework becomes more conservative in transmission and offloading, which reduces power usage but leads to a moderate increase in delay. This result demonstrates that the proposed sensor-aware MARL–ADMM framework can flexibly support different sensing applications with different service requirements. Therefore, the framework is applicable not only to low-latency sensor systems but also to energy-constrained monitoring scenarios requiring long-term and sustainable operation.

### 5.6. Discussion and Limitations

The comprehensive experimental evaluations conducted across various traffic scenarios and node scales demonstrate the strong efficacy and stability of the proposed MARL–ADMM framework. By compiling the empirical findings from both the baseline comparisons and statistical stability analyses, a clear synergistic performance paradigm emerges. While pure deep reinforcement learning baselines frequently suffer from severe resource violation and training instability under tight multi-access constraints, the coupling between local MARL inference and the edge ADMM consensus layer inherently bounds the network-wide delay and power consumption. The consistent superiority in total reward validates that introducing mathematical decomposition into multi-agent decision-making can effectively prevent chaotic resource mismatch and queue congestion under the considered simulation conditions.

From the perspective of simulation-based computational analysis, the computational footprint of this framework appears well-balanced within the evaluated scenario. On the client side, the computational overhead is extremely lightweight. Each sensor node only executes the decentralized forward inference of its local actor network, which is structurally configured as a basic multilayer perceptron (MLP). The per-step computational complexity scales as $O(\sum_{l}H_{v,l}H_{v,l+1})$ based on the hidden layer dimensions, requiring only standard matrix-vector multiplications that can be efficiently handled by low-power commercial microcontrollers within a few milliseconds with negligible power consumption. On the edge side, the computational complexity is governed by the centralized ADMM resource coordination loop. Since the dual updates are linear and the primal allocation subproblems yield analytical closed-form solutions, the coordination layer converges reliably within a deterministic maximum iteration bound $I_{max}$, exhibiting a practical complexity of $O(I_{max}\cdot NM)$ for *N* sensors and *M* access points. Hosted on dedicated edge servers with robust processing capabilities, this coordination achieves real-time scheduling responsiveness without draining the limited batteries of the sensory nodes.

To further quantify the practical feasibility, we report the measured execution time statistics under the baseline configuration. For offline training, the MARL component requires approximately 8.5 h. The training is performed on the edge server side and is a one-time cost before deployment. For online execution, the per-sensor decision time (actor–network forward pass) is measured at an average of 0.35 ms per decision slot on the sensor side, which is negligible compared with typical sensing-task processing delays. These statistics confirm that the proposed framework is computationally feasible for real-time deployment in resource-constrained edge-assisted WSNs.

Despite these advantages, several specific limitations of the current framework should be noted. First, the current model assumes relatively accurate system state information, whereas practical sensing environments inevitably involve estimation errors and dynamic reporting delays, which may lead to sub-optimal ADMM convergence. Moreover, the scalability of the MARL-based approach may be affected as the number of sensor nodes increases significantly, because the expanding joint state-action space increases the offline training time and memory footprint on the edge servers during the centralized training phase. Lastly, the framework adopts simplified sensing-task representations and does not explicitly incorporate domain-specific sensing-quality metrics such as data fidelity or information freshness.

## 6. Conclusions

This paper proposes a sensor-aware joint optimization framework for edge-assisted wireless sensor networks. The framework models devices as sensing nodes with heterogeneous task priorities, limited battery energy, bidirectional communication needs, and mission-oriented delivery requirements. Based on this framework, a MARL–ADMM solution is developed to jointly optimize sensing-task offloading, transmission power, and coordinated edge-resource allocation.

By embedding sensing semantics into system modeling, optimization objectives, and performance evaluation, the proposed framework provides a more realistic abstraction for intelligent sensor networks than conventional uplink-only MEC formulations. The simulation results demonstrate that the proposed method significantly reduces task delay, improves energy efficiency, and enhances the sustainability of sensing-network operation compared with several representative baselines in the evaluated scenario, indicating its potential for practical applications such as environmental monitoring, industrial sensing, intelligent inspection, and infrastructure surveillance.

Future work may incorporate explicit sensing-quality models, such as coverage fidelity, event-detection accuracy, or information freshness, and extend the current framework to mobile sensors, UAV-assisted relays, mobile sinks, and integrated sensing, communication, and computing scenarios. Although the proposed framework has been evaluated under different sensor-network scales and dynamic workload settings, the current validation remains simulation-based. Future work will further examine the proposed MARL–ADMM framework using real sensing datasets, embedded edge devices, and hardware testbeds, so as to assess the impact of practical factors such as hardware heterogeneity, protocol overhead, packet retransmission, and environmental interference.

## Figures and Tables

**Table 1:** Comparison between representative related studies and the proposed framework.

Study	WSN/IoT	Priority	Bidirectional	Power	Edge	Learning
	Scenario	Awareness	Traffic	Control	Coordination	Decision
Ma et al. [12]	Yes	No	No	Partial	Cloudlet-based	No
Chen et al. [13]	Yes	No	No	Yes	MEC-based	No
Zhao et al. [17]	No	No	No	Yes	Centralized	No
Qin et al. [11]	No	No	No	Yes	User-centric	Partial
Shen et al. [4]	Partial	Partial	Partial	Yes	Edge-assisted	DRL
Huang et al. [5]	Yes	Yes	Partial	Yes	ISCC-oriented	No
**Proposed framework**	Yes	Yes	Yes	Yes	MARL–ADMM	Yes

**Table 2:** Simulation Parameters

Parameter	Value
**Network Configuration**	
Number of sensor nodes	60
Number of APs	50
Number of CPUs	3
Sensor–node transmit power levels	{0,0.2,…,100} mW
AP downlink power budget	200 mW
AP computational capacity	[10, 15] GHz
CPU computational capacity	[15, 20] GHz
**Channel Model**	
Uplink carrier frequency	1.9 GHz
Downlink carrier frequency	28 GHz
Uplink bandwidth	2 MHz
Downlink bandwidth	2 GHz
Path loss exponents	2.5/3.8
Shadow fading STD	8/5.8 dB
**Algorithm Parameters**	
MARL learning rate	$5\times 10^{-4}$
Discount factor	0.99
PPO clip ratio	0.2
Actor–network architecture	MLP, 128–64, ReLU
Critic network architecture	MLP, 128–64, ReLU
PPO update epochs per iteration	10
PPO batch size	256
GAE parameter	0.95
Value-function coefficient	0.5
Entropy coefficient	0.01
Maximum gradient norm	0.5
ADMM penalty factor	1.0

**Table 3:** Statistical stability and reproducibility analysis over five independent runs (bold font indicates optimal results).

Method	Delay			Power Consumption			Reward		
	Mean ± Std	95% CI	p-Value	Mean ± Std	95% CI	p-Value	Mean ± Std	95% CI	p-Value
CBO	56.4582±0.3706	[55.9981,56.9184]	$1.632\times 10^{-12}$	42.3866±0.2492	[42.0771,42.6960]	$2.950\times 10^{-10}$	−46.1237±0.1940	[−46.3646,−45.8827]	$1.432\times 10^{-14}$
TRPO	51.3209±0.4008	[50.8232,51.8186]	$7.040\times 10^{-11}$	44.0431±0.2683	[43.7100,44.3763]	$3.427\times 10^{-10}$	−41.4406±0.4259	[−41.9695,−40.9118]	$3.801\times 10^{-9}$
MPO	63.4467±0.3205	[63.0488,63.8446]	$2.702\times 10^{-14}$	38.7943±0.1815	[38.5689,39.0196]	$5.246\times 10^{-11}$	−35.1568±0.2607	[−35.4805,−34.8331]	$6.100\times 10^{-10}$
LP	90.5817±0.1136	[90.4406,90.7228]	$2.841\times 10^{-12}$	49.0283±0.0875	[48.9196,49.1369]	$1.205\times 10^{-14}$	−58.7904±0.0957	[−58.9092,−58.6716]	$5.735\times 10^{-13}$
AO	105.3540±0.1928	[105.1146,105.5935]	$5.833\times 10^{-16}$	45.2632±0.0952	[45.1450,45.3813]	$2.748\times 10^{-14}$	−63.0558±0.1606	[−63.2552,−62.8565]	$1.687\times 10^{-16}$
SAO	44.0444±0.4090	[43.5366,44.5523]	$1.150\times 10^{-7}$	35.1505±0.2894	[34.7911,35.5098]	$9.268\times 10^{-6}$	−32.6888±0.3123	[−33.0766,−32.3010]	$3.139\times 10^{-7}$
MAPPO	40.4884±0.4737	[39.9002,41.0767]	$8.518\times 10^{-3}$	33.5255±0.1552	[33.3328,33.7182]	$4.631\times 10^{-4}$	−30.6658±0.2392	[−30.9628,−30.3688]	$4.412\times 10^{-5}$
MADDPG	41.0891±0.1809	[40.8645,41.3138]	$4.969\times 10^{-5}$	34.5844±0.1656	[34.3788,34.7901]	$2.755\times 10^{-7}$	−32.6035±0.3537	[−33.0426,−32.1643]	$1.432\times 10^{-6}$
Graph RL	42.4311±0.2702	[42.0956,42.7666]	$3.404\times 10^{-7}$	34.1372±0.0746	[34.0447,34.2298]	$1.882\times 10^{-6}$	−31.5605±0.3443	[−31.9880,−31.1330]	$1.485\times 10^{-5}$
Federated RL	43.2960±0.2615	[42.9713,43.6207]	$4.509\times 10^{-8}$	34.5518±0.1537	[34.3609,34.7426]	$1.760\times 10^{-7}$	−32.4924±0.4044	[−32.9946,−31.9902]	$6.776\times 10^{-6}$
**Ours**	**39.5673 ± 0.3089**	**[39.1837, 39.9509]**	–	**32.9995 ± 0.1328**	**[32.8346, 33.1643]**	–	**−29.5177 ± 0.2066**	**[−29.7742, −29.2612]**	–

**Table 4:** Ablation study of the proposed sensor-aware MARL–ADMM framework.

Method	Delay (ms)	Power (mW)	Reward
Full Model	39.5673±0.3089	32.9995±0.1328	−29.5177±0.2066
w/o ADMM	49.8412±0.6124	37.2854±0.2415	−37.9610±0.4105
w/o Downlink Modeling	47.1205±0.5218	35.9647±0.1985	−35.8423±0.3542
w/o Queue-Aware Reward	44.6391±0.4412	34.8812±0.1654	−33.7255±0.2874
w/o Task Priority	42.3704±0.3587	33.9458±0.1420	−31.8641±0.2314

**Table 5:** Sensitivity analysis with respect to the number of sensor nodes.

Number of Sensor Nodes	Delay (ms)	Power (mW)	Reward
20	35.8421±0.2145	31.7204±0.0954	−26.4812±0.1412
40	37.9150±0.2687	32.2415±0.1104	−27.8834±0.1854
60	39.5673±0.3089	32.9995±0.1328	−29.5177±0.2066
80	42.3782±0.3654	33.6219±0.1642	−31.7405±0.2415
100	45.9143±0.4412	34.4820±0.1985	−34.2981±0.2987

**Table 6:** Sensitivity analysis with respect to the sensing-task arrival rate.

Arrival Rate (Tasks/s/Node)	Delay (ms)	Power (mW)	Reward
0.4	35.4712±0.2014	31.9504±0.0984	−26.9142±0.1354
0.6	37.8256±0.2541	32.3618±0.1142	−28.1409±0.1742
0.8	39.5673±0.3089	32.9995±0.1328	−29.5177±0.2066
1.0	42.8804±0.3951	33.5742±0.1584	−32.0835±0.2514
1.2	46.7429±0.4874	34.4105±0.1912	−35.2618±0.3142

**Table 7:** Sensitivity analysis with respect to reward weights.

λ:μ	Delay (ms)	Power (mW)	Reward
0.7:0.3	38.2104±0.2874	33.6412±0.1542	−30.0418±0.2142
0.6:0.4	38.8751±0.2912	33.1804±0.1410	−29.7356±0.2087
0.5:0.5	39.5673±0.3089	32.9995±0.1328	−29.5177±0.2066
0.4:0.6	40.4823±0.3214	32.3150±0.1104	−29.8812±0.2104
0.3:0.7	41.7395±0.3451	31.9247±0.0924	−30.6754±0.2241

## Data Availability

The data presented in this study are available on request from the corresponding author.

## Abbreviations

WSN	Wireless sensor network
IoT	Internet of Things
MEC	Mobile edge computing
AP	Access point
MARL	Multi-agent reinforcement learning
ADMM	Alternating direction method of multipliers
PPO	Proximal policy optimization
TRPO	Trust region policy optimization
MPO	Maximum a posteriori policy optimization
